# Posterior reversible encephalopathy syndrome associated with use of anlotinib to treat squamous cell carcinoma of the cervix: case report and literature review

**DOI:** 10.3389/fphar.2023.1255785

**Published:** 2023-12-15

**Authors:** Jietao Lin, Wenmin Chen, Sha Zhong, Kai Qian, Hanrui Chen, Lizhu Lin

**Affiliations:** ^1^ The First Affiliated Hospital of Chinese Medicine, Guangzhou University of Chinese Medicine, Guagnzhou, Guangdong, China; ^2^ Baiyun Hospital of the First Affiliated Hospital of Guangzhou University of Chinese Medicine, Guagnzhou, Guangdong, China; ^3^ First Clinical Medical College, Guangzhou University of Chinese Medicine, Guangzhou, Guangdong, China

**Keywords:** posterior reversible encephalopathy syndrome, anlotinib, cervical carcinoma, targeted therapy, case report

## Abstract

**Background:** Posterior reversible encephalopathy syndrome (PRES), a neurological disorder with an unknown aetiology, is characterised by visual impairment, headache, vomiting, seizures, and transient alterations in consciousness.

**Case report:** We present the case of a 49-year-old woman with advanced cervical carcinoma who received second-line therapy with oral anlotinib (12 mg, days 1–14, every 21 days) and injectable tislelizumab (200 mg, day 1, every 21 days). After 7 days of anlotinib administration, she began experiencing symptoms suggestive of PRES and was diagnosed on day 11. Interruption of anlotinib and supportive treatment led to recovery of her binocular vision. The Naranjo score (+5) graded the causality of this reaction as probable, suggesting the possibility that the event may have been an adverse reaction to anlotinib.

**Ethics:** This case report was approved by the Ethics Committee of the First Affiliated Hospital of Guangzhou University of Traditional Chinese Medicine (Reference no. K-2023-068, 2023/06/09). Informed consent was obtained from the patient and her family.

## Highlights

1. PRES is a rare neurological disorder that typically develops after the use of certain drugs. We report a new case of anlotinib-induced PRES.
2. The pathogenesis of PRES and appropriate preventive strategies are unclear. Arterial hypertension (with BP increases beyond the upper brain blood pressure autoregulation limits) plays a pathophysiologically relevant, pivotal role in approximately 70%–80% of PRES cases. In order to share interesting information on PRES, we summarized the characteristics of similar cases in previous reports to provide useful clinical information.
3. Our patient experienced vision recovery after blindness for 48 h.

## 1 Introduction

Cervical cancer is the fourth most common malignant tumor in women. Furthermore, it is associated with significant health problems (Buskwofie et al., 2020). The worldwide incidence of cervical cancer is almost 13.1 cases per 100,000 women (Deo et al., 2022). Primary treatment modalities for cervical cancer are radiotherapy and surgery; chemotherapy, targeted therapy, and immunotherapy serve as supplementary treatments (Kumar et al., 2018). In recent years, the use of anti-angiogenesis targeted therapy and immune checkpoint inhibitors has emerged as a key focus of anti-tumor research, offering a new therapeutic avenue for management of advanced cervical cancer.

Anlotinib (Focus V^®^; Jiangsu Chia-Tai Tianqing Pharmaceutical Co., Ltd., Jiangsu, China) is a tyrosine kinase inhibitor that inhibits the phosphorylation of vascular endothelial growth factor receptors (e.g., VECFR, PDGFR, FGFR, and c-Kit); this blocks downstream signal transduction, prevents the formation of internal or peripheral blood vessels, and hinders tumor growth (Zirlik and Duyster, 2018). Anlotinib is effective in the treatment of various malignancies, such as lung, colorectal, thyroid, cervical, ovarian, and breast cancers (Zhu et al., 2021). In recent years, anlotinib has received substantial attention in the field of gynaecological oncology; several clinical trials have highlighted its effectiveness in the treatment of advanced cervical cancer.

Posterior reversible encephalopathy syndrome (PRES) is a relatively rare complication associated with the use of anti-angiogenic drugs. It is characterised by visual impairment, headache, vomiting, seizures, and altered consciousness (Parasher and Jhamb, 2020). Although blood pressure increases beyond the upper brain blood pressure autoregulation limits play a pivotal pathophysiological role in 70%–80% of PRES cases (Fugate and Rabinstein, 2015; Lava et al., 2017), the exact mechanism and aetiology of PRES is still not completely elucidated, and few reports have described its occurrence after the administration of anti-angiogenic agents. A search of the PubMed database revealed only two documented cases of PRES after treatment with anlotinib, an anti-tumor therapy (Nan et al., 2021; Zou et al., 2023). Here, we describe a patient with advanced squamous cell cervical cancer who developed PRES shortly after the initiation of anlotinib treatment. Fortunately, the patient’s vision rapidly recovered, and seizures were controlled after conventional treatment. Additionally, we summarize the clinical features, potential mechanisms, and therapeutic responses associated with PRES to provide insights for clinical treatment and prevention of PRES.

## 2 Case presentation

A 49-year-old woman was diagnosed with invasive low-differentiated squamous cell cervical cancer at our hospital in December 2021. She underwent radical resection at Sun Yat-sen University Cancer Hospital on 17 January 2022; postoperative pathological analysis and staging revealed a stage IIIC2. Over the next year, she received four cycles of adjuvant chemotherapy (paclitaxel liposome + cisplatin) and radiotherapy (2 Gy/25 cycles).

In January 2023, the patient noticed a firm, immobile lymph node in the left side of the neck, which was attached to the surrounding tissue. Computed tomography and lymph node biopsy confirmed tumor metastasis in the left supraclavicular nodes. Based on the Response Evaluation Criteria in Solid Tumors (RECIST v. 1.1), the patient was diagnosed with progressive disease. In February 2023, she began second-line therapy with oral anlotinib (12 mg, days 1–14, every 21 days) and intravenous tislelizumab (200 mg, day 1, every 21 days).

Seven days after initiation of anlotinib, the patient began experiencing persistent dizziness, headache, poor appetite, and elevated blood pressure (up to 200/100 mmHg). On 20 February 2023, she presented to the emergency department with sudden vision loss and limb twitching. Physical examination revealed that the patient was conscious without abnormalities in memory, orientation, or calculation. Her left and right pupils measured 4.5 and 3 mm, respectively, light reflexes were slightly sluggish. The hearing test yielded normal results. The frontal striae were symmetrical, eyelid closure was symmetrical and forceful, and nasolabial folds were symmetrical on both sides. The bilateral masseter muscles exhibited adequate strength, and the lower lip was centrally located upon mouth opening. Bilateral soft palate elevation was robust, and bilateral pharyngeal reflexes were present. The extended tongue was centrally located and did not exhibit any tremors. The limb muscle strength grade was 5, and limb muscle tension was normal. Abdominal, biceps, triceps, and knee reflexes were symmetrical and normal. Sensory localisation in the left upper limb was abnormal, whereas other parts of the body exhibited normal and symmetrical deep and superficial sensations. Neurological examination revealed a supple neck with negative Bruckner’s and Kirschner’s signs, as well as bilateral positive Babinski’s sign. A computed tomography angiography (CTA) of the head artery and the CTA of the carotid artery ruled out brain hernia, cerebral hemorrhage, and other emergencies. Furthermore, a brain magnetic resonance examination was requested.

**FIGURE 1:**
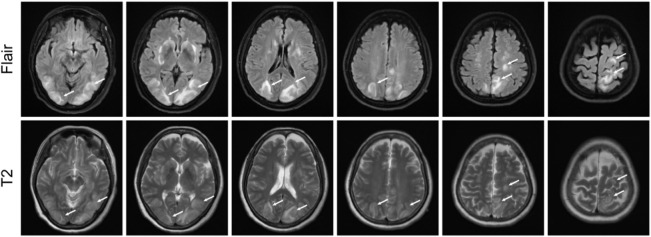
Magnetic resonance imaging results showing possible posterior reversible encephalopathy syndrome. T2 and fluid-attenuated inversion recovery images showing strong signals in bilateral frontal lobes, occipital lobes, and basal ganglia.

**FIGURE 2:**
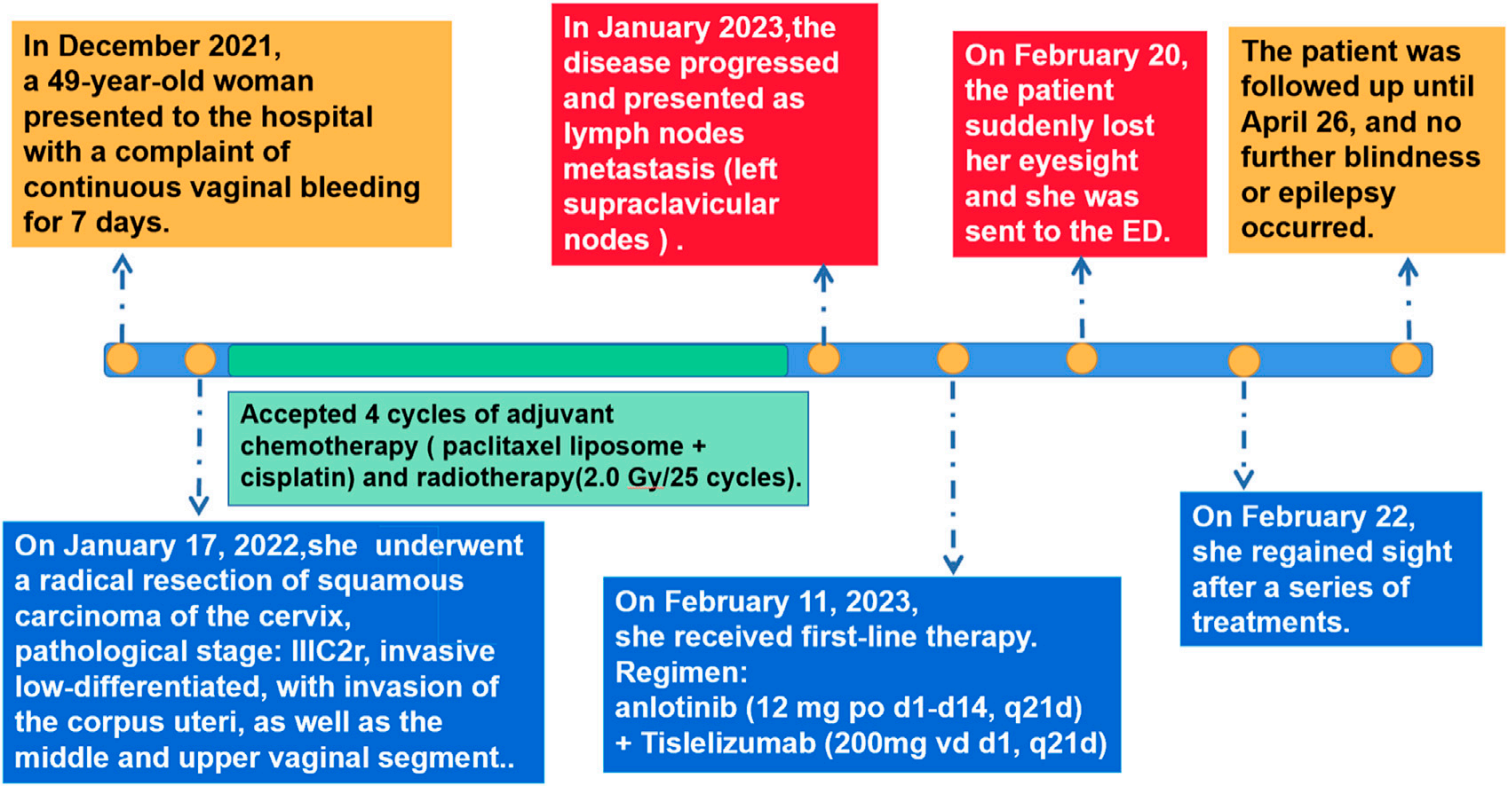
Diagnosis and treatment steps. ED, emergency department.

On 22 February 2023, cranial magnetic resonance imaging (MRI) showed multiple abnormal signals in the bilateral frontal lobes, occipital lobes, and basal ganglia, suggestive of PRES (Figure 1). The electroencephalogram (EEG) showed moderately abnormal brain activity. When the patient was conscious and quiet with her eyes closed, the EEG waves were bilaterally symmetrical; the main frequencies were in the θ-wave range (5–7 Hz; 30–60 µV) and α-wave range (8–10 Hz; 20–40 µV), with a few low-amplitude waves. Short bursts of β-wave activity (2–3.5 Hz; 30–80 µV) were also observed. During the eye-opening condition and eye-closing induction test, the α-wave was inhibited, whereas the EEG showed no obvious abnormality during hyperventilation and the flash stimulation test. Other blood tests (e.g., for liver and kidney functions, inflammatory markers, and blood levels) were normal. Based on the patient’s clinical symptoms and MRI findings, a diagnosis of PRES and seizures was made. Because of high blood pressure, the patient had already be instructed to discontinue anlotinib on February 18th. On the first day of blindness, the patient received intravenous injections of 0.4 g/dL sodium valproate and 10 mg/dL diazepam to control the seizures, 20 mg nifedipine sustained-release tablets to control the blood pressure, combined with an intravenous injection of 8 mg/dL betahistine hydrochloride to improve cerebral circulation and reduce systemic blood pressure. Furthermore, she received treatment for gastric mucosa protection, and nutritional support. Over the next few days, the patient also received intravenous injections of 0.5 g citicoline sodium and 20 mL/day Xingnaojing. The seizures were controlled on day 1 without recurrence. The symptoms gradually improved. On February 22, the patient exhibited no dizziness, headache or seizures; her binocular vision returned to normal. Because of economical constrains, brain imaging follow-up was limited to CT, which was performed on July 14 and showed that the brain line structure was centered, the brain cleft was not wide, and the size and shape of each ventricle were normal. In addition, no abnormal density lesions were found. She then received tislelizumab as maintenance therapy again and there was no recurrence of blindness or epilepsy. Figure 2 presents the patient’s diagnosis and treatment.

## 3 Discussion

Posterior reversible leukoencephalopathy syndrome (PRES) was initially described by Hinchey et al. (1996). It can be triggered by multiple factors, most commonly hypertensive encephalopathy, eclampsia, and the use of cytotoxic or immunosuppressive drugs (Garg RK, 2001; Stott et al., 2005). PRES was encountered in patients receiving bevacizumab in 2006 (Glusker et al., 2006). Since then, PRES has been described in patients receiving VEGF inhibitors, including sorafenib (Govindarajan et al., 2006; Laruelle et al., 2018), sunitinib (Kapiteijn et al., 2007), aflibercept (Leighl et al., 2010), regorafenib (Myint et al., 2014), pazopanib (Asaithambi et al., 2013), and anlotinib (Tlemsani et al., 2011; Kaneda et al., 2012). Furthermore, a Japanese patient developed PRES after treatment with the HER2 inhibitor trastuzumab. In that case, the pathogenesis may have been associated with the inhibition of tumor angiogenesis by trastuzumab, which reduced VEGF production and activated anti-angiogenic factors (Zhang et al., 2020).

There were several factors that supported a diagnosis of PRES in our patient. First, she had symptoms and physical examination findings suggestive of PRES, including blindness in both eyes, dizziness, headache, limb convulsions, normal superficial and deep sensations (except for the left upper limb), normal muscle strength and tension in both limbs, and bilateral positive Babinski’s sign. Second, MRI showed lesions in the basal ganglia, frontal and occipital lobes, and apex region. PRES should be distinguished from peripheral sensory disorders. Considering that sensitivity disorders typically occur after injury to the cerebral cortex, the presence of imaging evidence indicating brain injury in our patient, and her subsequent recovery of sensory loss, we believe that the sensitivity disorder in our patient’s left upper limb was caused by central nervous system damage, rather than peripheral nervous system damage.

A causative assessment through a structured algorithm revealed a Naranjo score of +5 (Table 1), suggesting a probable role of anlotinib as a cause (Naranjo et al., 1981). We believe that anlotinib may have been the trigger of PRES in this patient. Indeed, there were several factors supporting a potential causative role of anlotinib in the development of PRES in this patient. First, the patient had a history of anti-tumor drug use before symptom onset. Second, although she started tislelizumab and anlotinib therapy at about the same time, there are currently no reports of immunotherapy-induced PRES (tislelizumab belongs to this class of medications), and the same side effects did not occur when the patient was subsequently treated with tislelizumab again.

**TABLE 1:** ADR (Adverse drug reaction) probability scale.

To assess the adverse drug reaction, please answer the following questionnaire and give the pertinent score				
Questions	Yes	No	Don’t konw	Score
1. Are there previous conclusive reports on this reaction?	+1	0	0	+1
2. Did the adverse event appear after the suspected drug was administered?	+2	−1	0	+2
3. Did the adverse reaction improve when the drug was discontinued or a specific antagonist was administered?	+1	0	0	+1
4. Did the adverse reaction reappear when the drug was re-administered?	+2	−1	0	0
5. Are there alternative causes (other than the drug) that could on their own have caused the reaction?	−1	+2	0	0
6. Did the reaction reappear when a placebo was given?	−1	+1	0	0
7. Was the drug detected in the blood (or other fluids) in concentrations known to be toxic?	+1	0	0	0
8. Was the reaction more severe when the dose was increased, or less severe when the dose was decreased?	+1	0	0	0
9. Did the patient have a similar reaction to the same or similar drugs in any previous exposure?	+1	0	0	0
10. Was the adverse event confirmed by any objective evidence?	+1	0	0	+1
Total score				+5

Categorizations of the total score are associated with the degree of causality, with the score of ≥9 indicating definitity, 5–8 indicating probability, 1–4 indicating possibility, and ≤0 indicating doubt.

Although the pathogenesis of VEGF inhibitor-related PRES has not been fully elucidated, it likely involves damage to cerebral vascular endothelial cells (Hinchey et al., 1996). VEGF inhibitors can cause endothelial damage, thereby disrupting the blood-brain barrier and leading to increased perfusion, drug-induced vasospasm, oedema, and hypertension (Leighl et al., 2010).

Since its approval for clinical use in 2018, anlotinib has demonstrated a relatively good safety profile. The most commonly reported adverse events during anlotinib use include anaemia, hand-foot syndrome, leukopenia, leucocyturia, fatigue, haematuria, and hypertension (Kaneda et al., 2012; Asaithambi et al., 2013; Myint et al., 2014). Nan et al. (2021) (Tlemsani et al., 2011) found that rare adverse events associated with anlotinib use include myocardial infarction, hypertensive retinopathy, bronchopleural fistula, and esophago-tracheobronchial fistula.

We searched PubMed using the terms “posterior reversible encephalopathy syndrome and anlotinib,” “reversible posterior leukoencephalopathy syndrome and anlotinib,” and “epilepsy and anlotinib.” Our search revealed only two case reports regarding the development of PRES after anlotinib use (Nan et al., 2021; Zou et al., 2023). Furthermore, Zhang et al. (2020) described a patient who developed hypertensive retinopathy after anlotinib use. The patient experienced sudden painless vision loss in both eyes, accompanied by headache, nausea, and vomiting, similar to the symptoms of PRES. However, the patient did not undergo cerebral MRI and and was ultimately not diagnosed with PRES. Due to similar symptoms, we also included this case in the comparison below. (Table 2).

**TABLE 2:** Comparison of patients who developed confirmed or suspected PRES after anlotinib use.

	Publication year	Authors	Age/sex	Pathology type	Past illness	Dose	Highest recorded BP	Visual disturbance	Accompanying symptoms	Treatments	Prognosis
1	2020	Zhang et al. (2020)	48/F	Metastatic leiomyosarcoma	/	12 mg/3 months	164/109 mmHg	Yes	✓ Headache	✓ Angiotensin receptor blocker valsartan	Vision improved within 5 days but did not fully recover. A fluttering black shadow persisted 1 month later. No headache or vomiting. Blood pressure returned to normal
									✓ Nausea	✓ Circulation-promoting drug (Shuxuetong)	
									✓ Vomiting	✓ Nerve-nourishing drug (monosialotetrahexosylganglioside sodium)	
2	2021	Nan et al. (2021)	56/F	Lung adenocarcinoma	/	12 mg/5 months	217/120 mmHg	No	✓ Headache	✓ Intravenous urapidil, mannitol	No neurological deficits or hypertension Brain MRI revealed complete resolution of PRES findings compared with previous hospital admission
									✓ Vomiting		
									✓ Confusion	✓ Nifedipine	
3	2023	Zou et al. (2023)	37/F	Small cell lung cancer	Diabetes	10 mg/5 months, 8 mg/2.5 months	/	Yes	✓ Loss of appetite	/	Blurred vision did not resolve. No significant changes in brain MRI (white matter demyelination)
									✓ Weight loss		
									✓ Excessive fatigue		
4	2023	This case	49/F	Squamous carcinoma of the cervix	/	12 mg/7 days	200/100 mmHg	Yes	✓ Headache	✓ Rehydration and improved blood circulation	Complete recovery without hypertension or visual disturbance
									✓ Nausea	✓ Brain cell nourishment	
									✓ Vomiting	✓ Intracranial pressure reduction; seizure control	

F, female; mg, milligram; BP, blood pressure; mmHg, millimetres of mercury.

As shown in Table 2, all patients with confirmed or suspected PRES were women without a history of hypertension. Furthermore, all case reports were from China. Our patient had the shortest duration of anlotinib use among all patients, exhibited typical symptoms of PRES, and experienced rapid visual recovery after discontinuation of anlotinib.

The risk of PRES in patients receiving anti-VEGF drugs is unclear. Zou et al. (2023) searched PubMed for reports of PRES associated with anti-angiogenic therapy that were published between 2006 and 2023. The results showed that hypertension and increased serum levels of pro-inflammatory cytokines, including interleukin-6 and tumor necrosis factor-alpha, lead to endothelial damage or dysfunction in patients with PRES. In 2011, Tlemsani et al. (2011) analysed 28 reports of PRES in patients receiving anti-VEGF drugs (bevacizumab: *n* = 15, sunitinib: *n* = 7, sorafenib: *n* = 2, sunitinib and bevacizumab: *n* = 3, and aflibercept: *n* = 1). They found that most patients (73.1%) were women. Nearly one-third had a past history of hypertension. The most common symptoms included headache, visual impairment, and seizures. Most patients had hypertension at the time of PRES diagnosis, and all patients had albuminuria. Symptomatic treatment and appropriate blood pressure control led to favourable neurological outcomes Tlemsani et al. (2011). The risk of PRES is higher when blood pressure is poorly controlled and albuminuria is present. Additionally, hypomagnesemia may contribute to PRES onset (Shah, 2017; Zappia et al., 2020).

## Data Availability

The original contributions presented in the study are included in the article/Supplementary material, further inquiries can be directed to the corresponding authors.
